# Death from Chloroform

**Published:** 1861-01

**Authors:** 


### DEATH FROM CHLOROFORM.

P. C——, aged forty-two years, a tall, stout, muscular Irishman, was admitted, under the care of Mr. Mash, Sept. 1, 1860. He was the subject of a lipoma, about the size of a closed hand, seated in the middle line between the upper border of the scapulæ. On September 6th he was taken into the operating room for the purpose of having this removed. As the operation was considered neither dangerous nor very painful, he was strongly dissuaded by his surgeon from taking chloroform; but he, nevertheless, expressed an urgent desire that it should be administered. After consultation with Mr. Ashdown, as there seemed on auscultation to be no special contra-indicating circumstances, his wish was acceded to, and the chloroform was accordingly given by Mr. Gray, the house-surgeon. It was administered on a single fold of thin lint, and after three or four minutes' inhalation, the patient became excited and delirious, struggled a good deal, and talked incessantly. In the course of about eight minutes more these symptoms subsided, the face became much congested, and the breathing stertorous. At this time the pulse was full and regular, and the orbicular acted distinctly though rather sluggishly. The administration of chloroform was then wholly suspended, and the patient, being deemed in a fitting state for operation, was turned over on the left side in order to expose the tumor. This position being found inconvenient, a further change was required to adapt the head and shoulders on pillows, so as to expose the tumor effectually, and in the process of effecting this, respiration was observed suddenly to cease. The tongue was immediately drawn forward by artery forceps, cold water freely dashed over the face and chest, and artificial respiration was forthwith commenced by Marshall Hall's Method, aided by forcible compression of thoracic walls. At first the result of these means appeared most satisfactory, three or four deep muscular inspirations and several feebler efforts being made by the patient. The venous system of the face and neck being very turgid, the external jugular of either side was opened, and bled rather freely. Electro-magnetism also was applied as promptly as possible, the artificial respiration being unceasingly continued; but though the measures for restoring animation were continued for an hour, no further indications of vitality were manifested by the patient. No pulse or heart beat was detected after the first suspension of respiration, but the immediate resort to artificial breathing prevented any very deliberate examination of the pulse.

The chloroform seemed quite pure, and the total quantity consumed during the process of inhalation was nearly five drachms and a half. The patient was known to be intemperate, and has since been ascertained to have been an inveterate drinker.

*Autopsy twenty-three hours after death.*—Externally, the face appeared rather pale than livid (probably as a result of the venesection), and there was no more than usual ecchymosis of the depending portions of the body. The head was first opened. The dura mater was slightly adherent along the course of the longitudinal sinus; the glandulæ Pacchioni were of large size, and there was some subarachnoid effusion; but the membranes themselves appeared healthy. The substance of the brain was firm and natural; the puncta vasculosa were numerous and of a dark venous line. The lateral ventricles contained about two drachms of fluid; and the medulla oblongata and cerebellum, like the brain, were much congested, but presented no other evidences of disease. The lungs were gorged with venous blood, but otherwise healthy; the pleuræ smooth and unadherent. There was no pericardial effusion; the heart was large and flaccid, and there was an unusual accumulation of fat on its outer surface. Its muscular tissue was soft, and the walls of the ventricles, especially the left, were thinner than natural, but not otherwise unhealthy. The right chambers were greatly distended, the left comparatively empty. The blood was quite fluid throughout; the valves, endocardium, and vessels at the base were normal. The heart weighed ten pounds, the aorta and other vessels being cut off close to its base. There was an excessive deposit of fatal external to the abdominal walls, as also in the subperitoneal cellular tissue and throughout the great omentum. The abdominal viscera, like all the other organs, were greatly congested, but exhibited no further indications of disease.—*London Lancet.*

